# Bioactivity assessment of peptides derived from salted jellyfish (*Rhopilema hispidum*) byproducts

**DOI:** 10.1371/journal.pone.0318781

**Published:** 2025-02-11

**Authors:** Pratchaya Muangrod, Wiriya Charoenchokpanich, Sittiruk Roytrakul, Vilai Rungsardthong, Sawanya Charoenlappanit, Benjamaporn Wonganu, Lueacha Tabtimmai, Phumin Chamsodsai, Federico Casanova, Benjawan Thumthanaruk

**Affiliations:** 1 Department of Agro-Industrial, Food, and Environmental Technology, Faculty of Applied Science, King Mongkut’s University of Technology North Bangkok, Bangkok, Thailand; 2 Functional Proteomics Technology Laboratory, National Science and Technology Development Agency (NSTDA), Pathum Thani, Thailand; 3 Food and Agro-Industry Research Center, King Mongkut’s University of Technology North Bangkok, Bangkok, Thailand; 4 Center for Food Industry Innovation Technology, King Mongkut’s University of Technology North Bangkok, Bangkok, Thailand; 5 Department of Biotechnology, Faculty of Applied Science, King Mongkut’s University of Technology North Bangkok, Bangkok, Thailand; 6 Interdisciplinary Program in genetic Engineering and Bioinformatics, Graduate School, Kasetsart University, Bangkok, Thailand; 7 Research Group for Food Production Engineering, National Food Institute, Technical University of Denmark, Kongens Lyngby, Denmark; Shiraz University, IRAN, ISLAMIC REPUBLIC OF

## Abstract

The identification of multifunctional peptides derived from marine byproducts represents a significant challenge in the field. In Thailand, the fisheries industry exports salted jellyfish, which results in low-value byproducts primarily employed for animal feed. Previous studies have indicated the bioactivities of jellyfish protein hydrolysates from *Lobonema smitthii*; however, the multifunctional properties of *Rhopilema hispidum* remain largely unexplored. This research aimed to characterize synthetic bioactive peptides sourced from the byproducts of salted jellyfish (*R. hispidum*), with a specific emphasis on their antioxidant, angiotensin-I-converting enzyme (ACE) inhibitory, and anti-inflammatory activities. The hydrolysate obtained from the umbrella portion, subjected to pepsin treatment at a 3:20 enzyme-to-substrate ratio for 48 h at 37 °C, demonstrated the highest levels of antioxidant activity (DPPH =  1.85 ± 0.05 mM TE/mg protein, ABTS =  7.28 ± 0.03 mM TE/mg protein, FRAP =  3.04 ± 0.12 mM FeSO_4_/mg protein). Following purification, 18 novel peptides exhibiting high antioxidant scores (FRS+CHEL >  0.48) were identified and synthesized. Notably, the peptide MVVACVLPEA exhibited significant antioxidant (DPPH =  56.07 mM TE/mg protein), ACE inhibitory (91.69%), and anti-inflammatory activities (NO release =  34.59 µ M) without cytotoxic effects, although it is important to note that two other peptides did demonstrate cytotoxicity. This investigation reports a total of 16 synthesized peptides that possess triple functional activities—antioxidant, ACE inhibitory, and anti-inflammatory—without cytotoxicity, thus highlighting their potential applications in health-related fields.

## Introduction

Numerous human diseases, including diabetes, inflammatory conditions, cardiovascular diseases, and aging, are significantly influenced by oxidative stress. Implementing an effective antioxidation strategy to target free radicals is essential for disease prevention [1,2]. While inflammation acts as a defense mechanism against harmful stimuli, chronic inflammation can lead to various health complications [3]. Hypertension is the primary risk factor for serious cardiovascular events, such as atrial fibrillation and heart failure. The angiotensin-I-converting enzyme (ACE) plays a crucial role in regulating blood pressure by converting angiotensin I into angiotensin II, which induces vasoconstriction and raises blood pressure [4–10]. Persistently high blood pressure is a major contributor to cardiovascular diseases (CVDs) and is associated with premature mortality worldwide [1,7,11]. Targeted strategies that focus on antioxidation, inflammation reduction, and ACE inhibition are critical for effectively managing severe chronic diseases. Common ACE inhibitors, including enalapril and lisinopril, are widely used to treat hypertension but may have side effects such as cough and dizziness [1,7,11,12].

Research on natural inhibitors, particularly those derived from marine sources, has garnered substantial attention due to their minimal side effects and lack of residue accumulation [5]. Bioactive peptides stand out as effective and safe natural inhibitors, positioning themselves as strong alternatives to synthetic compounds [1]. These bioactive peptides consist of short amino acid chains, typically ranging from 2 to 20, and play critical roles in various biological processes that enhance human health [13]. Their efficient extraction often employs enzymatic hydrolysis, which effectively breaks down protein sources into smaller peptide fragments [1,14,15]. Different protein sources yield peptides with diverse and potent bioactivities, including antimicrobial, antioxidant, anti-inflammatory, and angiotensin-converting enzyme (ACE) inhibitory effects [13,16–18]. Marine organisms are recognized as exceptional sources of bioactive peptides, particularly those derived from shrimp, shellfish, crabs, and fish, which exhibit a wide range of beneficial properties [19]. For instance, antioxidant peptides have been definitively identified in miiuy croaker (*Miichthys miiuy*) [20], while ACE inhibitory peptides have been found in monkfish (*Lophius litulon*) swim bladders [21], and anti-inflammatory peptides have been isolated from sturgeon (*Acipenser schrenckii*) cartilage [22]. The biological activity of these peptides is significantly influenced by their size and amino acid sequence [17,18]. Lower molecular weight peptides, especially those enriched with hydrophobic amino acids, demonstrate notably enhanced activity [20–22]. Moreover, certain peptides exhibit multiple biological functions, which further underscores the necessity of exploring new marine protein sources.

Jellyfish have emerged as a compelling source for bioactive peptides. Studies unequivocally show that their protein hydrolysates deliver impressive antioxidants, ACE inhibitory, and antimicrobial properties [17,18,23–26]. Sand-type jellyfish (*R. hispidum*), an edible species, produces significant byproducts during salted processing; these byproducts, rich in protein, are ideally suited for peptide production [18]. While extant research has confirmed the antioxidant and antimicrobial properties of its hydrolysates [18], the anti-inflammatory and ACE inhibitory activities are areas ripe for further investigation.

The purification and characterization of bioactive peptides from sand-type jellyfish represents a novel and largely unexplored area of research. This study aimed to address this gap by purifying and identifying the bioactive peptides from the byproducts of sand-type salted jellyfish (*R. hispidum*). The identified peptides will subsequently undergo synthesis. Following synthesis, these peptides will be evaluated for their antioxidant activity, anti-inflammatory properties, ACE inhibitory effects, and cytotoxicity. This investigation will present the first detailed report on the multifunctional activities and cytotoxicity associated with peptide synthesis from sand-type salted jellyfish byproducts, providing a new perspective on the potential of marine bioactive peptides. The findings from this research could have significant implications for the treatment of severe chronic diseases. By showcasing the multifunctional activities and low cytotoxicity of marine bioactive peptides, this study may contribute to the development of safer and more effective treatments for conditions such as hypertension, inflammation, and oxidative stress.

## Materials and methods

### Materials and reagents

Salted jellyfish (*R. hispidum*) byproducts, such as broken or abnormal pieces, were received from Mahachai Food and Trading Co., Ltd., Samut Sakhon, Thailand. Pepsin (3000 NFU/mg) was purchased from HiMedia Laboratories Pvt. Ltd. (Nashik, India). 2,2 Diphenyl-1-Picryhydrazyl (DPPH), 2,2’-azino-bis (3-ethylbenzthiazoline-6-sulphonic acid) diammonium salt (ABTS), 2,4,6-Tris(2-pyridyl)-s-triazine (TPTZ), 6-hydroxy-2,5,7,8-tetramethylchroman-2-carboxylic acid (Trolox), and C18 spin column (Amberlite ^®^XAD^®^-2) were purchased from Sigma-Aldrich Co. (St. Louis, MO, USA). Cation exchange chromatography (SP SepharoseTM) and anion exchange chromatography (Q SepharoseTM) were purchased from Cytiva (Marlborough, MA, USA). Potassium persulfate was purchased from AppliChem GmbH (Darmstadt, Germany). ACE activity assay kit (Fluorometric) (catalog number CS0002) was purchased from Sigma-Aldrich Co. LLC. (Burlington, Massachusetts, US). The identified peptides were synthesized by GenScript Biotech Inc. (New Jersey, USA) (purity: > 85%). All materials were of analytical grade.

### Preparation of pepsin hydrolyzed jellyfish protein hydrolysate

The preparation of salted jellyfish byproducts was carried out using methods described in previous studies [17,18,27,28]. Initially, two samples (the umbrella and oral arms) were separated from the salted jellyfish byproducts. Each part was then washed with tap water using a washing machine, with a sample-to-water ratio of 1:10 (w/v), for two cycles of 15 min each to remove excess salt. After washing, the samples were drained for 30 min. They were then dried in a tray dryer (ED 400, Binder, USA) at 60 °C for 24 h. Once dried, the samples were crushed into small pieces and passed through a 100-mesh sieve. The resulting protein powder was stored in sealed polyethylene bags at room temperature until needed.

**Fig 1:**
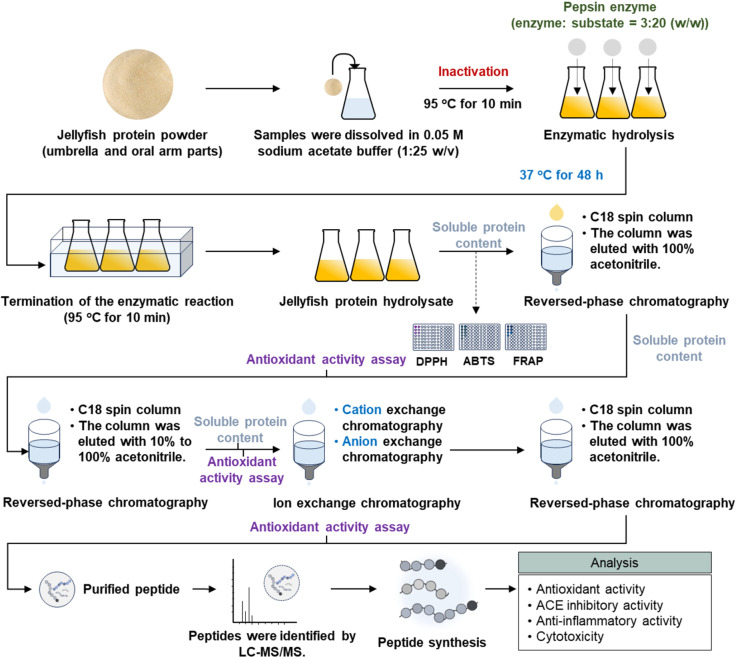
Research methodology flow chart.

Jellyfish protein hydrolysate was prepared with slight modifications to previous methods [18,29] (Fig 1). The jellyfish protein powder from the umbrella and oral arms was dissolved in 0.05 M sodium acetate buffer, using a ratio of 1:25 (w/v). The mixture was heated to 95 °C for 10 min to inactivate endogenous enzymes, then cooled, and pepsin enzyme was added at a ratio of 3:20 (enzyme: substrate, w/w). Hydrolysis was initiated by shaking the mixture at 37 °C and 150 rpm in a shaking incubator (WIS-20R, WiseCube, South Korea) for 48 h. Enzymatic hydrolysis was terminated by heating the mixture again at 95 °C for 10 min. The mixture was then centrifuged at 9,500 x g for 30 min and filtered through Whatman No. 1 filter paper. The resulting jellyfish protein hydrolysate was stored at -18 °C until analysis.

### Purification of peptides

The jellyfish protein hydrolysate was purified using reversed-phase and ion exchange chromatography techniques, specifically cation and anion exchange (Fig 1). Initially, 400 μL of hydrolysate was loaded into a C18 spin column (Amberlite ^®^XAD^®^-2) with a total column capacity of 1.0 mL, a length of 29.5 mm, and a diameter of 9 mm. The loaded columns were then flipped every 10 min for 2 h. After that, the columns were centrifuged at 1,000 rpm for 1 min. The column was washed with 400 μL of 0.1% formic acid 2 times and sterile water 1 time to remove impurities before being eluted with 400 μL of 100% acetonitrile 2 times. The eluent was then evaporated at 50 °C in a hot air oven. After drying, the sample was dissolved in a 10 mM acetate buffer and analyzed for soluble protein content and antioxidant activity. The sample was subsequently further purified using the C18 column, applying a stepwise elution of 10-100% acetonitrile in 10% increments. Antioxidant activity of all fractions was assessed, and those demonstrating activity were further purified using cation exchange chromatography (SP Sepharose^TM^) and anion exchange chromatography (Q Sepharose^TM^), both with a total column capacity of 0.9 mL, a length of 32 mm, and a diameter of 8 mm. A 1 M sodium chloride (NaCl) solution was used to elute the peptides from the column. The eluate from the cation and anion columns was then subjected to the C18 column to remove the NaCl, utilizing 100% acetonitrile as the eluent. The acetonitrile was subsequently removed using a hot air oven set to 50 °C. After evaporation, the remaining sample was dissolved in a 10 mM sodium acetate buffer at pH 4.0 and analyzed for soluble peptide content and antioxidant activity. This activity was evaluated using several assays: 2,2-diphenyl-1-picrylhydrazyl (DPPH), 2,2’-azino-bis (3-ethylbenzthiazoline-6-sulphonic acid) diammonium salt (ABTS), and ferric reducing antioxidant power (FRAP).

### Peptide synthesis

Peptides with an antioxidant activity score greater than 0.48 were synthesized by GenScript Biotech Inc. in New Jersey, USA, using the L-isomers of each amino acid by solid-phase synthesis with the fluorenylemthoxycarbonyl (Fmoc) method [30]. The purity of the synthesized peptides exceeded 85%, as assessed by high-performance liquid chromatography (HPLC) analysis.

### Cell culture

Murine macrophage cells (RAW 264.7; ATCC TIB-71™) were cultured in Dulbecco’s Modified Eagle’s Medium (DMEM) that was supplemented with 10% heat-inactivated fetal bovine serum (FBS) and 1% antibiotic solution. Colorectal adenocarcinoma cells (CaCo-2; ATCC HTB 37™) were cultured in Eagle’s Minimum Essential Medium (EMEM), also supplemented with 10% heat-inactivated FBS and 1% antibiotic solution. Both cell types were maintained in a humidified incubator with 5% CO_2_. The cell culture medium was refreshed every three days.

### Analysis

#### Determination of soluble protein content.

The soluble protein content of all samples was analyzed using the Lowry method [27]. Bovine serum albumin (BSA) served as the standard.

#### DPPH radical scavenging assay.

The DPPH radical scavenging activity of the sample was assessed following a modified protocol based on a previous study [18]. In each well of a 96-well plate, 10 μL of the sample was added, followed by 190 μL of 0.1 mM DPPH solution in 70% ethanol. The mixture was then incubated at room temperature in the dark for 30 min. After the incubation period, the absorbance of the mixture was measured at 517 nm. The DPPH radical scavenging activity was calculated using the following equation (1):


$$DPPHradicalscavengingactivity\left(\%\right)=\left[\left(A_{control}–A_{sample}\right)/A_{control}\right]\times 100$$ (1)

In this equation, *A*_*control*_ represents the absorbance of a control solution containing all reagents except for the test samples, while *A*_*sample*_ is the absorbance of the mixture (the sample combined with 0.1 mM DPPH). To determine the Trolox equivalent antioxidant capacity (TEAC), a Trolox standard curve was utilized. The results were expressed as millimoles of Trolox equivalents per milligram of protein (mM TE/mg protein).

#### ABTS radical scavenging assay.

The ABTS radical scavenging activity of the sample was measured using a method adapted from Mirzaei et al. [1]. The ABTS radical was generated by mixing 7 mM ABTS with 2.45 mM potassium persulfate in a 1:1 ratio and incubating the mixture in the dark at room temperature for 12 to 16 h. The resulting solution was then diluted to achieve an absorbance of 0.74 ± 0.03 at 734 nm, using deionized water for adjustments. Next, 10 μL of the sample was added to 190 μL of the ABTS solution. The mixture was incubated in the dark for 7 min, after which the absorbance was measured at 734 nm. The ABTS radical scavenging activity (%) was calculated using the following equation (2):


$$ABTSradicalscavengingactivity\left(\%\right)=\left[\left(A_{control}–A_{sample}\right)/A_{control}\right]\times 100$$ (2)

In this equation, *A*_*control*_ refers to the absorbance of the control solution (which contains all reagents except the test samples), while *A*_*sample*_ refers to the absorbance of the mixture containing the sample and the ABTS solution. To determine the Trolox equivalent antioxidant capacity (TEAC), a Trolox standard curve was used, and the results were expressed as millimoles of Trolox equivalents per milligram of protein (mM TE/mg protein).

#### Ferric reducing antioxidant power (FRAP) assay.

The FRAP assay was conducted following the method outlined by Rumpf et al. [31]. The FRAP solution was prepared by mixing 300 mM acetate buffer (pH 3.6), 10 mM TPTZ (2,4,6-Tris(2-pyridyl)-s-triazine) in 40 mM hydrochloric acid, and 20 mM FeCl_3_ in a ratio of 10:1:1. In a 96-well plate, 10 μL of the sample was combined with 190 μL of the FRAP solution and incubated in the dark for 15 min. The absorbance of the mixture was then measured at 593 nm. FeSO_4_ was used to create the standard curve, and the results were expressed as millimoles of FeSO_4_ equivalent per milligram of protein (mM FeSO_4_/mg protein).

#### Identification of purified peptides by LC-MS/MS.

The peptides were desalted through overnight dialysis against distilled water and subsequently analyzed using a Hybrid Quadrupole Q-TOF Impact II™ (Bruker Daltonics, Germany) combined with an Ultimate 3000 Nano/Capillary LC System (Thermo Scientific, USA). A total of 100 ng of peptide was loaded onto a µ -Precolumn (300 μm i.d. ×  5 mm C18 Pepmap 100, 5 μm, 100 Å, Thermo Scientific, UK) and then separated on a C18 reversed-phase column (15 cm in length ×  75 μm in diameter) packed with Acclaim PepMap1 RSLC C18 (3 μm, 100 Å, nanoViper, Thermo Scientific, USA). The column was maintained in a thermostat column oven set to 40 °C. Solvents A and B, consisting of 0.05% formic acid in water and 0.1% formic acid in 80% acetonitrile respectively, were used in the analytical column. A linear gradient ranging from 5% to 55% of solvent B was applied to elute the peptides at a flow rate of 300 μL/min over 30 min, following equilibration with solvent A. Electrospray ionization was performed at 1.6 kV using the CaptiveSpray, with nitrogen serving as the drying gas at a flow rate of approximately 50 L/h. Collision-Induced Dissociation (CID) product ion mass spectra were generated using nitrogen gas as the collision gas. Mass spectra (MS) and MS/MS spectra were recorded over a mass-to-charge ratio (m/z) range of 150–2200 utilizing Compass 1.9 software from Bruker Daltonics. The collision energy was adjusted to 10 eV according to the m/z value.

#### Prediction of antioxidant activities.

The antioxidant activities of the peptides, specifically their free radical scavenging (FRS) and ion chelating (CHEL) capabilities, were predicted using a convolutional neural network within the AnOxPePred software (https://services.healthtech.dtu.dk/services/AnOxPePred-1.0/). The results are expressed as a score ranging from 0 to 1, where a score closer to 1 indicates a higher effectiveness as an antioxidant. Additionally, the isoelectric point (pI) and net charge of the peptide were determined using PepDraw software.

#### ACE inhibitory activity assay.

The ACE inhibitory activity assay was performed following the method outlined by Batista et al. [32], with slight modifications, using a fluorometric angiotensin I-converting enzyme (ACE) activity assay kit from Sigma-Aldrich (catalog number CS0002), Burlington, Massachusetts, USA. In summary, 10 µ L of the sample (1 mg/mL for the peptide sample and 50 µg/mL for ramipril) was combined with 40 µ L of the ACE solution (CS0002B) in 96-well flat-bottom black plates at 37 °C for 5 min. Next, 50 µ L of the substrate solution (CS0002C) was added to this mixture to initiate the reaction. A fluorescence multiwell plate reader was used to monitor the changes in fluorescence. The sample’s fluorescence was measured every min for 5 min, with excitation at 320 nm and emission at 405 nm. All reagents, except the test sample, were prepared to assess the ACE positive control activity. A kit standard solution (CS0002D) was diluted with assay buffer (CS0002A) to create different concentrations (nmol) of the standard solution, allowing generation of a standard curve. The slope of the standard curve (RFU/nmol) and the slope of the sample kinetic curve were utilized to calculate the ACE activity using the following equation (3):


$$ACEactivity\left(nmol/min\right)=\left(Slope_{sample}/Slope_{standard}\right)\times DF$$ (3)

Where *Slope*_*sample*_ refers to the slope of the blank-subtracted sample curve (RFU/min), *Slope*_*standard*_ refers to the slope of the blank-subtracted standard curve (RFU/nmol), *DF* is the dilution factor, and 0.1 nmol/min is equivalent to 0.1 units or 100 mU.

In this study, the results of ACE inhibitory activity are expressed as percentages. The ACE inhibitory activity (%) was calculated using the following equation (4):


$$ACEinhibitoryactivity\left(\%\right)=\left[\left(ACE_{PC}–ACE_{S}\right)/ACE_{PC}\right]\times 100$$ (4)

Where *ACE*_*PC*_ represents the ACE activity of the positive control, and *ACE*_*S*_ denotes the ACE activity of the sample.

#### Anti-inflammation assay.

The anti-inflammation assay was conducted following the method described by Barzkar et al. [33] with slight modifications. RAW 264.7 (1 x 10^5^ cells) was seeded into a 96-well plate to allow for cell attachment. After 24 h, the cells were pre-treated with fresh medium containing peptide samples at a concentration of 10 µg/mL for 1 h. Subsequently, the treated cells were stimulated with 1 µg/mL lipopolysaccharide (LPS) for an additional 24 h. Unstimulated cells were included as a negative control. At the end of the treatment period, the conditioned medium was collected for the determination of released nitric oxide (NO). Griess’s reagent (Promega) was utilized to measure the NO concentration, following the manufacturer’s instructions. A NO calibration curve was generated to quantify the released NO in the conditioned medium.

#### Cytotoxicity assessment.

Cytotoxicity assessment was performed following the method described by Yang et al. [2], with slight modifications. RAW 264.7 was seeded with 1 x 10^5^ cells in a 96-well plate with an appropriate cell culture medium. After allowing the cells to attach, the medium was replaced with a fresh cell culture medium containing the peptide samples at a concentration of 10 µg/mL. The treated cells were incubated for an additional 72 h. At the designated time, fresh medium was added, which included 3-(4,5-dimethylthiazol-2-yl)-2,5-diphenyltetrazolium bromide (MTT) at a concentration of 0.5 mg/mL. After 3 h, formazan crystals were observed. The medium was then removed, and the crystals were dissolved in 100% dimethyl sulfoxide (DMSO). A microplate reader (Synergy HTX; Biotek, Multi-Mode Reader, Winooski, VT, USA) was utilized to measure the absorbance at 570 nm (measurement wavelength) and 630 nm (reference wavelength). The percentage of cell viability was calculated relative to untreated cells using the following equation (5):


$$Cellviability\left(\%\right)=\left[\left(OD_{treatment}–OD_{blank}\right)/\left(OD_{untreated}–OD_{blank}\right)\right]\times 100$$ (5)

Where *OD*_*treatment*_ is the absorbance of the treated sample, *OD*_*untreated*_ is the absorbance of the untreated sample, and *OD*_*blank*_ is the absorbance from wells without treated and untreated samples.

### Statistical analysis

All experiments were conducted in triplicate (n  = 3). Results are presented as mean±standard deviation. Statistical analysis was performed using the Statistical Package for the Social Sciences (SPSS) version 22.0. Duncan’s multiple range test was utilized to evaluate significant differences among the compared groups. A p-value of less than 0.05 was considered statistically significant.

## Results and discussion

### Antioxidant activity of salted jellyfish byproducts protein hydrolysate

The soluble protein content of protein hydrolysates derived from jellyfish umbrella (PU) and oral arms (PO) parts, which were hydrolyzed with pepsin at a ratio of 3:20 w/w for 48 h at 37 °C, was found to be 14.42 ± 0.33 mg/mL for PU and 13.64 ± 0.45 mg/mL for PO (S1 Table). This soluble protein content is higher than that of white-type jellyfish hydrolyzed with pepsin at a ratio of 1:20 w/w for 24 h at 37 °C, which yielded a range of 10.06–11.56 mg/mL [18]. The difference in soluble protein content can be attributed to factors such as the type of raw materials used, collagen types, the activity of the pepsin during hydrolysis, and the duration of the hydrolysis process. Furthermore, pepsin hydrolysis disrupts the collagen triple-helix structure by cleaving collagen protein chains at specific amino acid sites, resulting in smaller molecular weight and size proteins compared to the original collagen [34]. Consequently, the soluble protein content tends to increase with a higher pepsin concentration and a longer hydrolysis time.

In terms of antioxidant activity, the DPPH radical scavenging activity of PU and PO was 20.19 ± 2.86% (TEAC =  1.85 ± 0.05 mM TE/mg protein) and 16.92 ± 0.89% (TEAC =  1.57 ± 0.10 mM TE/mg protein), respectively (S2 Table). When compared to jellyfish protein hydrolysates reported in previous studies, the percentage of DPPH inhibition in the protein hydrolysates increased with longer hydrolysis times and greater use of pepsin. Specifically, the DPPH radical scavenging activities of PU (20.19%) and PO (16.92%) were higher than those of jellyfish protein hydrolysates, which ranged from 8.4% to 10.02%. This comparison was made after the protein hydrolysates were treated with pepsin at a substrate-to-enzyme ratio of 1:20 w/w for 24 h. Increasing the pepsin content and extending the hydrolysis time produces greater amounts of hydrolyzed jellyfish protein, resulting in hydrolysates that contain more short-chain amino acids. Xia et al. [35] reported that the antioxidant activity of peptides is influenced by their structure, amino acid composition, sequence, and molecular weight. Additionally, protein hydrolysates that consist of shorter amino acid chains (ranging from 2 to 20 amino acids) tend to demonstrate more potent biological activity. Guo et al. [36] have also noted that low molecular weight hydrolysates and peptides exhibit high antioxidant activity. Consequently, jellyfish protein hydrolysate treated with pepsin at a ratio of 3:20 w/w for 48 h displayed higher DPPH radical scavenging activity than those treated with pepsin at a 1:20 w/w ratio for 24 h. Ambigaipalan and Shahidi [37] reported that the DPPH and ABTS radical scavenging activities of shrimp shell protein hydrolysate were measured at 0.01 mM TE/g sample and 0.03 mM TE/g sample, respectively. In contrast, Guo et al. [38] found that the protein hydrolysate from armoured catfish (*Pterygoplichthys disjunctivus*) exhibited an ABTS radical scavenging activity of 0.17 mM TE/g sample. Additionally, Upata et al. [26] reported that the FRAP of jellyfish (*L. smithii*) protein hydrolysate ranged from 0.24 to 0.65 mM FeSO_4_/g sample. The variability in bioactivity can be attributed to the different methods and enzymes used for hydrolysis, as well as the quantity and types of amino acids present in each sample. Notably, sand-type jellyfish contains a relatively high amount of hydrophobic amino acids. When hydrolyzed with pepsin, jellyfish protein produces short peptide chains that are rich in hydrophobic amino acids, which positively influence the bioactivity of the peptides. Consequently, the jellyfish protein hydrolysate was subjected to purification in the subsequent step.

Furthermore, the ABTS radical scavenging activity of PU and PO was recorded at 7.28 ± 0.03 and 7.23 ± 0.12 mM TE/mg protein (S2 Table), respectively. The ferric reducing antioxidant power (FRAP) of PU and PO was measured at 3.04 ± 0.12 and 2.05 ± 0.15 mM FeSO_4_/mg protein (S2 Table), respectively. Overall, the jellyfish protein hydrolysate hydrolyzed with pepsin for 48 h in this study exhibited higher antioxidant activity compared to protein hydrolysates derived from other marine sources.

### Effect of purification on antioxidant activity of peptide fractions from jellyfish protein hydrolysate

The hydrolysates of PU and PO were purified and designated as PUR and POR. The DPPH radical scavenging activities of PUR and POR were 3.60 ± 0.30 and 2.51 ± 0.18 mM TE/mg protein, respectively. Additionally, the ABTS radical scavenging activities for PUR and POR were found to be 11.10 ± 0.33 and 9.10 ± 0.31 mM TE/mg protein, respectively. Furthermore, the ferric reducing antioxidant power (FRAP) values for PUR and POR were recorded as 3.35 ± 0.35 and 2.58 ± 0.33 mM FeSO_4_/mg protein (S3 Table), respectively. These results indicate that the purified fractions exhibited higher antioxidant activity than the unpurified ones. This increase in activity can be attributed to the protein hydrolysate’s higher hydrophobicity and lower molecular weight following purification by reversed-phase chromatography. These findings align with previous research, which suggests that peptides with greater hydrophobicity and lower molecular weight tend to demonstrate enhanced antioxidant activity compared to more hydrophilic and heavier peptides [39]. Hydrophobic amino acids in a peptide chain improve solubility in lipids, making the peptides more accessible to radical species [39]. The jellyfish peptides (PUR and POR) were further subjected to gradient purification using acetonitrile at varying concentrations (10–100%), leading to fractions labeled as PUR10-PUR100 and POR10-POR100. The results showed that increasing the concentration of acetonitrile from 10% to 50% resulted in the following soluble protein contents for PUR10-PUR50: 1.03 ± 0.04, 2.09 ± 0.14, 1.42 ± 0.04, 0.37 ± 0.02, and 0.01 ± 0.00 mg/mL, respectively. Similarly, the soluble protein contents for POR10-POR50 were 0.92 ± 0.04, 2.08 ± 0.09, 1.38 ± 0.01, 0.40 ± 0.10, and 0.05 ± 0.01 mg/mL (S4 Table). Selected peptide fractions (PUR10, PUR20, PUR30, POR10, POR20, POR30) were then analyzed for antioxidant activity due to their high soluble protein content, which was sufficient for further testing.

The antioxidant activity (measured by DPPH, ABTS, and FRAP assays) of PUR and POR, following purification through reversed-phase chromatography and elution with varying concentrations of acetonitrile (10-30%), is detailed in Table 1 and S5 Table. The jellyfish peptide fractions eluted with 20% acetonitrile (designated as PUR20 and POR20) demonstrated the highest scavenging activity for DPPH and ABTS radicals, as well as enhanced FRAP activity. Specifically, the antioxidant activity of the jellyfish peptide fractions eluted with 20% acetonitrile showed an increase of 2.24 to 2.75 times in DPPH and ABTS radical scavenging activity, and an increase of 1.77 to 1.91 times in FRAP activity, compared to the antioxidant activity of the jellyfish protein hydrolysate before purification. Consequently, the jellyfish peptide fractions PUR20 and POR20 were selected for further purification using cation and anion exchange chromatography.

**Table 1:** The antioxidant activity (DPPH, ABTS, FRAP) of jellyfish peptide after purification by reversed-phase chromatography (10–30% acetonitrile =  eluent).

Sample		Antioxidant activity		
		DPPH (mM TE/mg protein)	ABTS (mM TE/mg protein)	FRAP (mM FeSO_4_/mg protein)
**10%ACN**	**PUR**	3.15 ± 0.30^BC^	10.10 ± 0.20^B^	2.58 ± 0.12^C^
	**POR**	2.69 ± 0.39^C^	9.37 ± 0.11^C^	2.30 ± 0.21^C^
**20%ACN**	**PUR**	5.10 ± 0.37^A^	13.17 ± 0.33^A^	5.39 ± 0.64^A^
	**POR**	3.52 ± 0.30^B^	10.26 ± 0.17^B^	3.92 ± 0.94^B^
**30%ACN**	**PUR**	3.08 ± 0.12^BC^	10.14 ± 0.05^B^	2.44 ± 0.24^C^
	**POR**	1.89 ± 0.34^D^	9.30 ± 0.13^C^	1.74 ± 0.32^C^

Different superscripts (A, B, C, and D) in the same column mean a significant difference in value (p < 0.05), ACN =  Acetonitrile.

PUR20 and POR20 were purified using ion exchange chromatography (both cation and anion) and were labeled as PUR20C, POR20C, PUR20A, and POR20A. The antioxidant activities of these peptides, measured using DPPH, ABTS, and FRAP assays, are presented in Table 2 and S6 Table. All purified peptide fractions from both cation and anion exchange chromatography demonstrated the highest ABTS radical scavenging activity, with PUR20C and POR20C showing greater values than PUR20A and POR20A. A similar trend was observed in DPPH radical scavenging activities, although the values were much lower. In contrast, the FRAP activity of these peptide fractions was the lowest, with anion exchange chromatography fractions exhibiting higher values than those from cation exchange chromatography. When comparing the umbrella and oral arms of the jellyfish, the results indicated that the umbrella part consistently had higher antioxidant activities, regardless of the purification method or antioxidant analysis used. The peptide fraction purified by cation-exchange chromatography contains numerous cationic amino acids, which negatively affect the samples’ ferric-reducing antioxidant power. These findings are consistent with a previous study that reported a decrease in FRAP activity as the amount of cationic amino acids in the sample increased [40]. Based on the highest observed antioxidant activity, the PUR20C sample was selected for peptide sequence identification.

**Table 2:** The antioxidant activity (DPPH, ABTS, FRAP) of jellyfish peptide after purification by ion exchange chromatography (cation and anion exchange chromatography) and reverse phase chromatography (C18 column).

Sample		Antioxidant activity		
		DPPH (mM TE/mg protein)	ABTS (mM TE/mg protein)	FRAP (mM FeSO_4_/mg protein)
**Cation**	**PUR20**	5.52 ± 0.06^A^	14.17 ± 0.11^A^	1.81 ± 0.12^C^
	**POR20**	3.66 ± 0.17^B^	11.03 ± 0.42^B^	0.76 ± 0.12^D^
**Anion**	**PUR20**	2.48 ± 0.31^C^	7.15 ± 0.42^C^	2.86 ± 0.12^A^
	**POR20**	1.36 ± 0.11^D^	5.58 ± 0.47^D^	2.23 ± 0.12^B^

Different superscripts (A, B, C, and D) in the same column mean a significant difference in value (p < 0.05).

### Peptide sequence identification

The amino acid sequence and molecular weight of the most active peptide (PUR20C) were determined using LC-MS/MS. Eighteen identified peptides, namely NQKAMQELNE, TDSPAPSETTD, EIILIPMF, EQIYPMGEGDEL, HSVLTASYRN, RARVVPMY, EIDKLPPESRP, RPLAKIIP, GKNCGETVWE, NFTVLCDSLE, LNIPKPKLNLL, LMFMAVAFF, LGAEGRSP, PFTMYFLL, KFNEVVYFL, MVVACVLPEA, PMETDDQPNN, and DAENKENVEE, had molecular weights ranging from 785.40 to 1379.59 Da and contained between 8 and 12 amino acid residues (Table 3). These peptides with an antioxidant activity score (FRS +  CHEL) greater than 0.48, were selected for synthesis.

**Table 3:** Peptides identified from jellyfish protein hydrolysate purified by reversed-phase (C18 column) and cation exchange chromatography.

Code	Peptide sequence	m/z	Charge	pI	ALC	FRS	CHEL
P1	NQKAMQELNE	1,203.55	−1	4.08	51.99	0.28	0.20
P2	TDSPAPSETTD	1,119.45	−3	2.76	50.60	0.35	0.28
P3	EIILIPMF	974.55	−1	3.09	48.50	0.37	0.25
P4	EQIYPMGEGDEL	1,379.59	−4	2.73	47.82	0.42	0.19
P5	HSVLTASYRN	1,146.57	+1	9.40	47.08	0.35	0.22
P6	RARVVPMY	990.54	+2	11.15	46.37	0.39	0.20
P7	EIDKLPPESRP	1,279.67	−1	4.33	45.13	0.40	0.26
P8	RPLAKIIP	906.60	+2	11.71	44.97	0.32	0.21
P9	GKNCGETVWE	1,121.48	−1	4.09	43.24	0.37	0.17
P10	NFTVLCDSLE	1,139.51	−2	2.89	43.24	0.33	0.20
P11	LNIPKPKLNLL	1,261.81	+2	10.64	32.00	0.33	0.25
P12	LMFMAVAFF	1,075.52	0	5.52	29.57	0.38	0.22
P13	LGAEGRSP	785.40	0	7.16	29.38	0.38	0.20
P14	PFTMYFLL	1,030.52	0	5.22	28.51	0.51	0.19
P15	KFNEVVYFL	1,157.61	0	6.75	26.60	0.39	0.18
P16	MVVACVLPEA	1,030.51	−1	3.10	25.73	0.37	0.18
P17	PMETDDQPNN	1,159.44	−3	2.69	24.92	0.34	0.23
P18	DAENKENVEE	1,175.49	−4	3.45	22.74	0.33	0.21

pI =  isoelectric point, ALC =  average local confidence, FRS =  free radical scavenging, CHEL =  ion chelating, P =  peptide.

### Bioactivity of synthetic peptides

#### Antioxidant activity of synthetic peptides.

**Fig 2:**
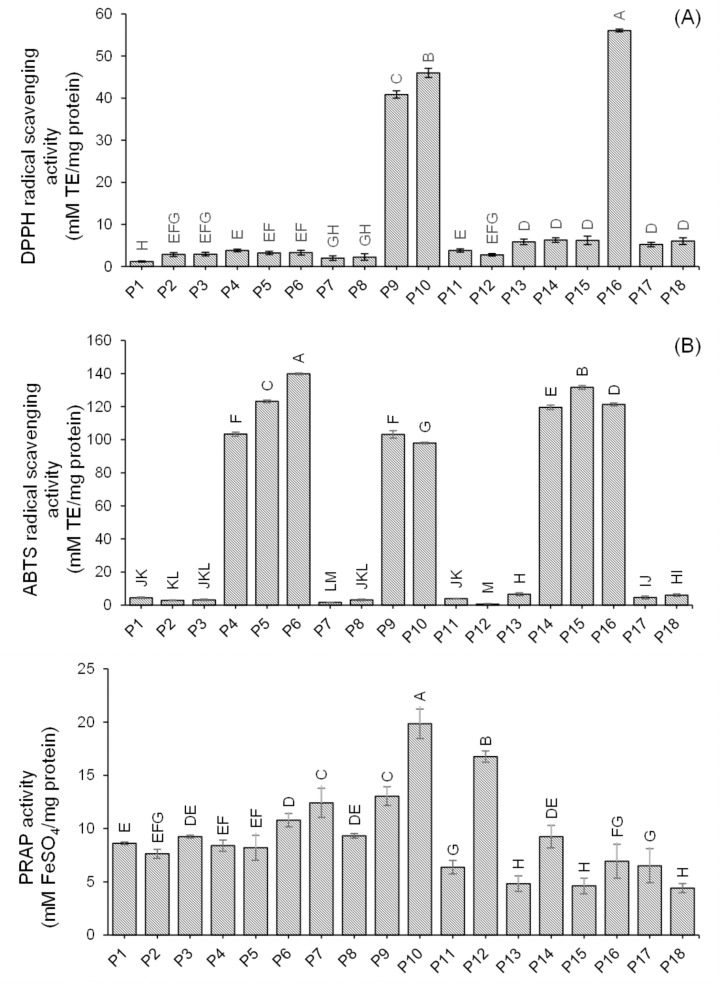
Antioxidant activity; (A) DPPH radical scavenging activity(B) ABTS radical scavenging activity, and (C) FRAP activity of synthetic peptides. Bars with the different letters in the same activity indicate statistically significant differences (p < 0.05).

These 18 synthesized peptides (P1-P18) were tested for their antioxidant properties, which included DPPH radical scavenging activity, ABTS radical scavenging activity, and FRAP activity. The results indicated that the DPPH radical scavenging activity varied from 1.16 to 56.07 mM TE/mg protein, the ABTS radical scavenging activity ranged from 0.64 to 139.87 mM TE/mg protein, and the FRAP activity was between 4.40 and 19.84 mM FeSO_4_/mg protein (Fig 2 and S7 Table). Compared to previous studies [17,25,36,37], these peptides demonstrated higher antioxidant activity. The antioxidant potential of the peptides is influenced by factors such as molecular weight, structure, amino acid composition, and sequence.

Peptide P16 (MVVACVLPEA) displayed the strongest DPPH radical scavenging activity due to its non-polar or hydrophobic composition, which includes methionine, valine, alanine, leucine, and proline. These peptides can act as antioxidants because they contain many electrons that can neutralize free radicals by donating electrons [41]. The presence of hydrophobic and aromatic amino acids significantly impacts the antioxidant potential of the peptides. Additionally, cysteine and glutamic acid in P16 contribute to its potent antioxidant activity because it can donate electrons to free radicals. For ABTS radical scavenging activity, peptide P6 (RARVVPMY) exhibited the highest activity, attributed to the presence of tyrosine, an amino acid containing a phenolic group known for its strong antioxidant properties. Regarding FRAP activity, peptide P10 (NFTVLCDSLE), which contains cysteine, showed the highest activity due to the electron donation ability of certain amino acid residues, including the sulfhydryl group of cysteine.

#### ACE inhibitory activity of synthetic peptides.

**Fig 3:**
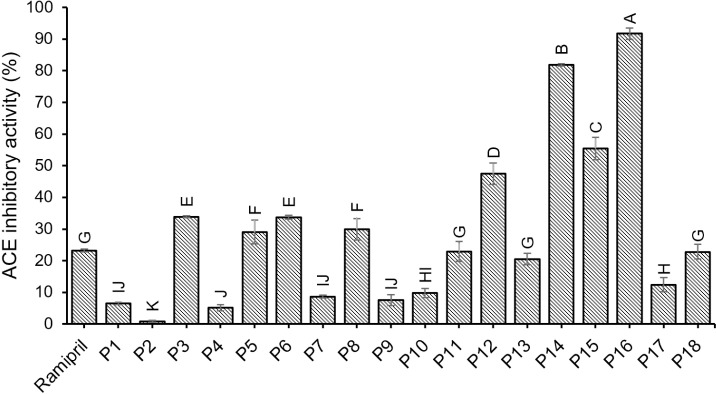
ACE inhibitory activity of synthetic peptides. Bars with the different letters indicate statistically significant differences (p < 0.05).

The ACE inhibitory activity of synthetic peptides was evaluated using an ACE activity assay kit. The results were expressed as a percentage of ACE inhibitory activity, as illustrated in Fig 3. The findings showed that the ACE inhibitory activity of ramipril (an ACE inhibitor) was 23.23 ± 0.44%, while the ACE inhibitory activity of peptides P1 to P18 ranged from 0.85% to 91.69% (S8 Table). Among these, peptide P16 demonstrated the highest ACE inhibitory activity at 91.69 ± 1.78%, with a molecular weight of 1.03 kDa. This high activity may be attributed to the presence of hydrophobic amino acids—methionine, valine, alanine, leucine, and proline—in the P16 peptide sequence (MVVACVLPEA), particularly with proline situated at the C-terminal tripeptide positions.

Research indicates that the ACE inhibitory activity of peptides depends on the peptide’s structural features specific to the active site of ACE. Peptides act as inhibitors that have a similar structure to the substrate and bind to the active site of the enzyme, preventing the substrate from binding to the active site of ACE, thus helping to inhibit or reduce the activity of ACE [42]. The amino acid at the C-terminal position of the peptide is important for the ACE inhibitory activity. The ACE inhibitory activity of the peptide is enhanced when the C-terminal position of the peptide contains non-polar and hydrophobic amino acids or has non-polar and hydrophobic amino acids attached to the amino acid branch at the C-terminal position [42]. Additionally, short-chain peptides also bind the active site of enzymes more easily than long-chain peptides, which allows them to increase their bioactivity more than long-chain peptides [42]. The previous study highlighted that the amino acid composition at the C-terminal tripeptide positions plays a significant role in determining the ACE inhibitory activity of specific peptides derived from Pacific saury [43]. In P16, proline and alanine are located at the C-terminal tripeptide and C-terminal positions, while methionine, valine, alanine, and proline are found in the peptide chain, all of which positively impact its ACE inhibitory activity. Consequently, the MVVACVLPEA peptide (P16) was identified as a potent ACE inhibitory peptide.

#### Anti-inflammatory activity of synthetic peptides.

**Fig 4:**
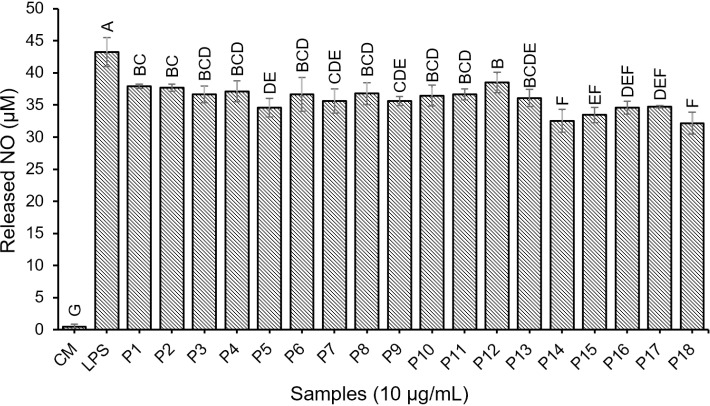
Anti-inflammatory activity of synthetic peptides. Bars with the different letters indicate statistically significant differences (p < 0.05).

The anti-inflammatory activity of synthetic peptides was assessed by measuring the concentration of nitric oxide (NO) they released, as NO levels are directly proportional to inflammation in the body. The results, shown in Fig 4, indicated that all synthetic peptides significantly released lower levels of NO compared to the control (43.21 ± 2.24 µ M) (S9 Table). Peptides P5 (HSVLTASYRN), P14 (PFTMYFLL), P15 (KFNEVVYFL), P16 (MVVACVLPEA), P17 (PMETDDQPNN), and P18 (DAENKENVEE) exhibited the most notable reductions, with a decrease in NO concentration ranging from 19.67% to 25.52% compared to the control. It was observed that peptides composed of hydrophobic amino acids demonstrated strong anti-inflammatory activity. Peptides that contain a high amount of hydrophobic amino acids can prevent LPS-stimulated inflammatory responses by binding to LPS to form peptide-lipopolysaccharide complexes and scavenging LPS by inducing cell membrane charge reversal [44]. Peptides containing hydrophobic amino acids at both the N-terminal and C-terminal positions showed pronounced anti-inflammatory effects [22]. Additionally, positively charged amino acids, such as lysine, arginine, and histidine, are crucial for enhancing the anti-inflammatory activity of peptides [44]. As described above, the type and the arrangement of amino acids within the peptide chain are all important for the anti-inflammatory effect. For instance, the presence of proline at the N-terminal and leucine at the C-terminal in the P14 (PFTMYFLL) or the presence of lysine at the N-terminal and leucine at the C-terminal in P15 (KFNEVVYFL) was associated with significant anti-inflammatory activity. These findings suggest that synthetic peptides derived from jellyfish protein hydrolysate possess anti-inflammatory properties and act as inhibitors of NO production.

#### Cytotoxicity of synthetic peptides.

**Fig 5:**
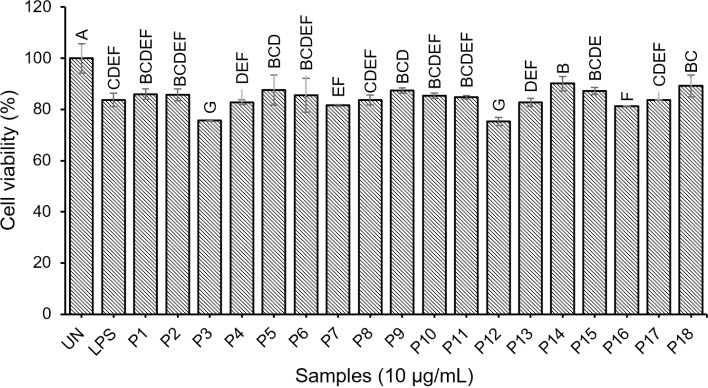
Cytotoxicity of synthetic peptides. Bars with the different letters indicate statistically significant differences (p < 0.05). UN =  untreated sample.

The cell viability of sample RAW264.7 was assessed using the MTT assay to evaluate the cytotoxic effects of synthetic peptides on cells. The results of the cytotoxicity analysis are illustrated in Fig 5. The percentage of cell survival for the synthetic peptides ranged from 75.27% to 90.09% (S10 Table). Notably, all synthetic peptides exhibited cell viability percentages exceeding 80%, except for P3 and P12, which showed lower survival rates. Previous research reported that the percentage of cell viability is lower than 80% compared with the control; the test sample is cytotoxic [45]. Moreover, Ahrens et al. [46] reported that peptides are generally considered safe because they have low immunogenicity and produce non-toxic metabolites. This suggests that the synthetic peptides typically did not have significant cytotoxic effects on the cells.

#### The chemical structure and 3D structure of MVVACVLPEA.

**Fig 6:**
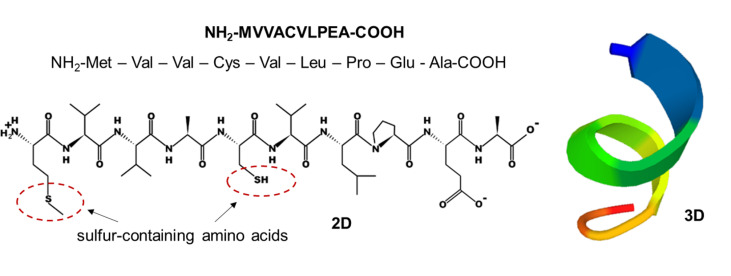
The chemical structure of MVVACVLPEA (P16). The peptide’s 2D structure obtained using the PepDraw tool, and the peptide’s 3D structure obtained using the PEP-FOLD3 tool.

The chemical structure and 3D structure of MVVACVLPEA (P16) are depicted in Fig 6. P16 has been shown to possess multiple bioactivities, including antioxidant, ACE inhibitory, and anti-inflammatory activities, while demonstrating no cytotoxic effects. An analysis of the secondary structure of P16 reveals that it has an alpha helix conformation. This peptide consists of 10 amino acids, each containing an amino group (NH_2_), a carboxyl group (COOH), and a specific R group (side chain) that determines its characteristics, such as size, polarity, and pH. The bioactivities of peptides can be influenced by the type, position, and quantity of amino acids within the peptide sequence [17,18]. Notably, 8 out of the 10 amino acids in P16 are hydrophobic, which positively impacts its antioxidant, ACE inhibitory, and anti-inflammatory activities. Additionally, P16 contains sulfur-containing amino acids, namely methionine and cysteine, which can react with free radicals. The presence of proline in the C-terminal tripeptide positions of P16 also enhances its ACE inhibitory activity. Furthermore, methionine at the N-terminal position and alanine at the C-terminal position significantly contribute to the potent anti-inflammatory activity of P16.

## Conclusions

Byproducts derived from salted sand-type jellyfish (*R. hispidum*) have the potential to serve as a source of bioactive peptides through the process of enzymatic hydrolysis. The peptide fraction obtained following purification via reversed-phase and cation exchange chromatography exhibited enhanced antioxidant activity compared to jellyfish protein hydrolysate. Among the 18 identified peptides, MVVACVLPEA (P16) demonstrated significant bioactivity, characterized by antioxidant, ACE inhibitory, and anti-inflammatory properties, while not exhibiting cytotoxic effects. Consequently, peptides obtained from sand-type jellyfish protein hydrolysate present multifunctional benefits as bioactive compounds. Nevertheless, additional research is required to scale up the production of jellyfish peptides and assess their applications within the biomedical and food sectors. Furthermore, the bioactivity of these peptides warrants investigation through in vivo studies, as they may undergo degradation and modification within the intestinal and vascular systems. Therefore, it is imperative to examine the correlation between in vitro and in vivo peptide activity.

## Supporting information

S1 Table The soluble protein content of protein hydrolysates derived from jellyfish umbrella (PU) and oral arms (PO) parts.
Different superscripts (A and B) in the same column mean a significant difference in value (p < 0.05).
(DOCX)

S2 Table The antioxidant activity (DPPH, ABTS, FRAP) of PU and PO.
Different superscripts (A and B) in the same column mean a significant difference in value (p < 0.05). ns =  Not significant (p < 0.05).
(DOCX)

S3 Table The antioxidant activity (DPPH, ABTS, FRAP) of PUR and POR.
Different superscripts (A and B) in the same column mean a significant difference in value (p < 0.05).
(DOCX)

S4 Table The soluble protein content of PUR10-PUR50 and POR10-POR50.
Different superscripts (A, B, C, D, and E) in the same column mean significant difference in value (p < 0.05). Different superscripts (a and b) in the same row mean significant difference in value (p < 0.05). ns =  Not significant (p < 0.05).
(DOCX)

S5 Table The antioxidant activity (DPPH, ABTS, FRAP) of jellyfish peptide after purification by reversed-phase chromatography (10-30% acetonitrile =  eluent).
Different superscripts (A, B, C, and D) in the same column mean a significant difference in value (p < 0.05). ACN =  Acetonitrile.
(DOCX)

S6 Table The antioxidant activity (DPPH, ABTS, FRAP) of jellyfish peptide after purification by ion exchange chromatography (cation and anion exchange chromatography) and reverse phase chromatography (C18 column).
Different superscripts (A, B, C, and D) in the same column mean a significant difference in value (p < 0.05).
(DOCX)

S7 Table The antioxidant activity (DPPH, ABTS, FRAP) of synthetic peptides (P1-P18).
Different superscripts (A, B, C, D, E, F, G, H, I, J, K, L, and M) in the same column mean a significant difference in value (p < 0.05).
(DOCX)

S8 Table The ACE inhibitory activity of synthetic peptides (P1-P18).
Different superscripts (A, B, C, D, E, F, G, H, I, J, and K) in the same column mean a significant difference in value (p < 0.05).
(DOCX)

S9 Table The anti-inflammatory activity of synthetic peptides (P1-P18).
Different superscripts (A, B, C, D, E, F, and G) in the same column mean a significant difference in value (p < 0.05).
(DOCX)

S10 Table Cytotoxicity of synthetic peptides (P1-P18). Different superscripts (A, B, C, D, E, F, G, and H) in the same column mean a significant difference in value (p < 0.05). UN =  untreated sample.
(DOCX)

## Abbreviations

ACE: Angiotensin-I-converting enzyme
RAS: Renin-angiotensin system
CVDs: Cardiovascular diseases
DPPH: (2,2-diphenyl-1-picrylhydrazyl
ABTS: 2,2’-azino-bis (3-ethylbenzthiazoline-6-sulphonic acid) diammonium salt
FRAP: Ferric reducing antioxidant power
TEAC: Trolox equivalent antioxidant capacity
FRS: Free radical scavenging
CHEL: Ion chelating
LPS: Lipopolysaccharide
NO: Nitric oxide
PU: The jellyfish protein hydrolysate from an umbrella part
PO: The jellyfish protein hydrolysate from oral arms part
PUR: PU after purification with reversed-phase chromatography
POR: PO after purification with reversed-phase chromatography
PUR10-PUR100: PUR after purification with reversed-phase chromatography and eluted with acetonitrile at 10-100%
POR10-POR100: POR after purification with reversed-phase chromatography and eluted with acetonitrile at 10-100%
PUR20C: PUR20 after purification with cation exchange chromatography
POR20C: POR20 after purification with cation exchange chromatography
PUR20A: PUR20 after purification with anion exchange chromatography
POR20A: POR20 after purification with anion exchange chromatography

## References

[1] Mirzaei M, Mirdamadi S, Ehsani MR, Aminlari M, Hosseini E. Purification and identification of antioxidant and ACE-inhibitory peptide from *Saccharomyces cerevisiae* protein hydrolysate. J Funct Foods. 2015;19:259–68. doi: 10.1016/j.jff.2015.09.031

[2] Yang J, Hu L, Cai T, Chen Q, Ma Q, Yang J, et al. Purification and identification of two novel antioxidant peptides from perilla (*Perilla frutescens* L. Britton) seed protein hydrolysates. PLoS One. 2018;13(7):e0200021. doi: 10.1371/journal.pone.0200021 29985955 PMC6037370

[3] Khursheed M, Ghelani H, Jan RK, Adrian TE. Anti-inflammatory effects of bioactive compounds from seaweeds, bryozoans, jellyfish, shellfish and peanut worms. Mar Drugs. 2023;21(10):524. doi: 10.3390/md21100524 37888459 PMC10608083

[4] Heo J-H, Kim E-A, Kang N, Heo S-Y, Ahn G, Heo S-J. The antioxidant effects of trypsin-hydrolysate derived from abalone viscera and fishery by-products, and the angiotensin-i converting enzyme (ACE) inhibitory activity of its purified bioactive peptides. Mar Drugs. 2024;22(10):461. doi: 10.3390/md22100461 39452868 PMC11509546

[5] Wu J, Liao W, Udenigwe CC. Revisiting the mechanisms of ACE inhibitory peptides from food proteins. Trends Food Sci Technol. 2017;69:214–9. doi: 10.1016/j.tifs.2017.07.011

[6] Beltrami L, Zingale LC, Carugo S, Cicardi M. Angiotensin-converting enzyme inhibitor-related angioedema: how to deal with it. Expert Opin Drug Saf. 2006;5(5):643–9. doi: 10.1517/14740338.5.5.643 16907654

[7] Zhang L, Miao J, Guo J, Liu J, Xia Z, Chen B, et al. Two novel angiotensin I-converting enzyme (ACE) inhibitory peptides from rice (*Oryza sativa* L.) bran protein. J Agric Food Chem. 2023;71(9):4153–62. doi: 10.1021/acs.jafc.2c07270 36812450

[8] Dou B, Liu Y, Liu Y, Fan L, Ma Y, Shi Y. Isolation and characterization of angiotensin I converting enzyme (ACE) inhibitory peptides from rice bran proteins and evaluation of activity and stability. PJZ. 2020;52(4):1383–91. doi: 10.17582/journal.pjz/20190416040458

[9] He Z, Liu G, Qiao Z, Cao Y, Song M. Novel angiotensin-I converting enzyme inhibitory peptides isolated from rice wine lees: purification, characterization, and structure-activity relationship. Front Nutr. 2021;8:746113. doi: 10.3389/fnut.2021.746113 34568409 PMC8460919

[10] Nuchprapha A, Paisansak S, Sangtanoo P, Srimongkol P, Saisavoey T, Reamtong O, et al. Two novel ACE inhibitory peptides isolated from longan seeds: purification, inhibitory kinetics and mechanisms. RSC Adv. 2020;10(22):12711–20. doi: 10.1039/d0ra00093k 35492113 PMC9051311

[11] Shi A, Liu H, Liu L, Hu H, Wang Q, Adhikari B. Isolation, purification and molecular mechanism of a peanut protein-derived ACE-inhibitory peptide. PLoS One. 2014;9(10):e111188. doi: 10.1371/journal.pone.0111188 25347076 PMC4210216

[12] Zheng W, Tian E, Liu Z, Zhou C, Yang P, Tian K, et al. Small molecule angiotensin converting enzyme inhibitors: A medicinal chemistry perspective. Front Pharmacol. 2022;13:968104. doi: 10.3389/fphar.2022.968104 36386190 PMC9664202

[13] Ktari N, Ben Slama-Ben Salem R, Bkhairia I, Ben Slima S, Nasri R, Ben Salah R, et al. Functional properties and biological activities of peptides from zebra blenny protein hydrolysates fractionated using ultrafiltration. Food Biosci. 2020;34:100539. doi: 10.1016/j.fbio.2020.100539

[14] Xia Y, Bamdad F, Gänzle M, Chen L. Fractionation and characterization of antioxidant peptides derived from barley glutelin by enzymatic hydrolysis. Food Chem. 2012;134(3):1509–18. doi: 10.1016/j.foodchem.2012.03.063 25005974

[15] Senadheera TRL, Hossain A, Dave D, Shahidi F. Antioxidant and ACE-inhibitory activity of protein hydrolysates produced from Atlantic sea cucumber (*Cucumaria frondosa*). Molecules. 2023;28(13):5263. doi: 10.3390/molecules28135263 37446924 PMC10343221

[16] Monari S, Ferri M, Russo C, Prandi B, Tedeschi T, Bellucci P, et al. Enzymatic production of bioactive peptides from scotta, an exhausted by-product of ricotta cheese processing. PLoS One. 2019;14(12):e0226834. doi: 10.1371/journal.pone.0226834 31887121 PMC6936807

[17] Muangrod P, Charoenchokpanich W, Rungsardthong V, Vatanyoopaisarn S, Wonganu B, Roytrakul S, et al. Effect of pepsin hydrolysis on antioxidant activity of jellyfish protein hydrolysate. E3S Web Conf. 2021;302:02010. doi: 10.1051/e3sconf/202130202010

[18] Muangrod P, Charoenchokpanich W, Roytrakul S, Rungsardthong V, Vatanyoopaisarn S, Charoenlappanit S, et al. Effect of pepsin on antioxidant and antibacterial activity of protein hydrolysate from salted jellyfish (*Lobonema smithii* and *Rhopilema hispidum*) by-products. E3S Web Conf. 2022;355:02013. doi: 10.1051/e3sconf/202235502013

[19] Mao Z, Jiang H, Sun J, Zhao Y, Gao X, Mao X. Research progress in the preparation and structure-activity relationship of bioactive peptides derived from aquatic foods. Trends Food Sci Technol. 2024;147:104443. doi: 10.1016/j.tifs.2024.104443

[20] Zhao W-H, Luo Q-B, Pan X, Chi C-F, Sun K-L, Wang B. Preparation, identification, and activity evaluation of ten antioxidant peptides from protein hydrolysate of swim bladders of miiuy croaker (*Miichthys miiuy*). J Funct Foods. 2018;47:503–11. doi: 10.1016/j.jff.2018.06.014

[21] Hu Y-D, Xi Q-H, Kong J, Zhao Y-Q, Chi C-F, Wang B. Angiotensin-I-converting enzyme (ACE)-inhibitory peptides from the collagens of monkfish (*Lophius litulon*) swim bladders: isolation, characterization, molecular docking analysis and activity evaluation. Mar Drugs. 2023;21(10):516. doi: 10.3390/md21100516 37888451 PMC10608021

[22] Yuan L, Chu Q, Yang B, Zhang W, Sun Q, Gao R. Purification and identification of anti-inflammatory peptides from sturgeon (*Acipenser schrenckii*) cartilage. Food Sci Hum Wellness. 2023;12(6):2175–83. doi: 10.1016/j.fshw.2023.03.030

[23] Lv Z, Zhang C, Song W, Chen Q, Wang Y. Jellyfish collagen hydrolysate alleviates inflammation and oxidative stress and improves gut microbe composition in high-fat diet-fed mice. Mediators Inflamm. 2022;2022:5628702. doi: 10.1155/2022/5628702 35979013 PMC9377926

[24] De Domenico S, De Rinaldis G, Paulmery M, Piraino S, Leone A. Barrel jellyfish (*Rhizostoma pulmo*) as source of antioxidant peptides. Mar Drugs. 2019;17(2):134. doi: 10.3390/md17020134 30813405 PMC6410228

[25] Barzideh Z, Latiff AA, Gan C-Y, Abedin MZ, Alias AK. ACE inhibitory and antioxidant activities of collagen hydrolysates from the ribbon jellyfish (*Chrysaora* sp.). Food Technol Biotechnol. 2014;52(4):495–504. doi: 10.17113/ftb.52.04.14.3641 27904323 PMC5079151

[26] Upata M, Siriwoharn T, Makkhun S, Yarnpakdee S, Regenstein JM, Wangtueai S. Tyrosinase Inhibitory and antioxidant activity of enzymatic protein hydrolysate from jellyfish (*Lobonema smithii*). Foods. 2022;11(4):615. doi: 10.3390/foods11040615 35206090 PMC8871577

[27] Charoenchokpanich W, Rungsardthong V, Vatanyoopaisarn S, Thumthanaruk B, Tamaki Y. Salt reduction in salted jellyfish (*Lobonema smithii*) using a mechanical washing machine. Sci Eng Health Stud. 2020;14(3):184–92. doi: 10.14456/sehs.2020.17

[28] Muangrod P, Rungsardthong V, Vatanyoopaisarn S, Tamaki Y, Kuraya E, Thumthanaruk B. Effect of wash cycle on physical and chemical properties of rehydrated jellyfish by-products and jellyfish protein powder. Science, Engineering and Health Studies. 2021;15:21030004. doi: 10.14456/sehs.2021.14

[29] Theeraraksakul K, Jaengwang K, Choowongkomon K, Tabtimmai L. Exploring the biological functions and anti-melanogenesis of *Phallus indusiatus* for mushroom-based cosmetic applications. Cosmetics. 2023;10(5):121. doi: 10.3390/cosmetics10050121

[30] Hansen PR, Oddo A. Fmoc solid-phase peptide synthesis. Methods Mol Biol. 2015;1348:33–50. doi: 10.1007/978-1-4939-2999-3_5 26424261

[31] Rumpf J, Burger R, Schulze M. Statistical evaluation of DPPH, ABTS, FRAP, and Folin-Ciocalteu assays to assess the antioxidant capacity of lignins. Int J Biol Macromol. 2023;233:123470. doi: 10.1016/j.ijbiomac.2023.123470 36736974

[32] Batista D, Chiocchetti G de ME, Macedo JA. Effect of enzymatic biotransformation on the hypotensive potential of red grape pomace extract. Foods. 2023;12(22):4109. doi: 10.3390/foods12224109 38002167 PMC10670604

[33] Barzkar N, Bunphueak P, Chamsodsai P, Muangrod P, Thumthanaruk B, Rungsardthong V, et al. Jellyfish protein hydrolysates: Multifunctional bioactivities unveiled in the battle against diabetes, inflammation, and bacterial pathogenesis. Microb Pathog. 2024;191:106648. doi: 10.1016/j.micpath.2024.106648 38641070

[34] severin S, Xia WS. Enzymatic hydrolysis of whey proteins by two different proteases and their effect on the functional properties of resulting protein hydrolysates. J Food Biochem. 2006;30(1):77–97. doi: 10.1111/j.1745-4514.2005.00048.x

[35] Xia J, Song H, Huang K, Li S, Guan X. Purification and characterization of antioxidant peptides from enzymatic hydrolysate of mungbean protein. J Food Sci. 2020;85(6):1735–41. doi: 10.1111/1750-3841.15139 32468582

[36] Guo P, Qi Y, Zhu C, Wang Q. Purification and identification of antioxidant peptides from Chinese cherry (*Prunus pseudocerasus* Lindl.) seeds. J Func Foods. 2015;19:394–403. doi: 10.1016/j.jff.2015.09.003

[37] Ambigaipalan P, Shahidi F. Bioactive peptides from shrimp shell processing discards: antioxidant and biological activities. J Func Foods. 2017;34:7–17. doi: 10.1016/j.jff.2017.04.013

[38] Guo Y, Michael N, Fonseca Madrigal J, Sosa Aguirre C, Jauregi P. Protein hydrolysate from *Pterygoplichthys disjunctivus*, armoured catfish, with high antioxidant activity. Molecules. 2019;24(8):1628. doi: 10.3390/molecules24081628 31027188 PMC6514753

[39] Khositanon P, Panya N, Roytrakul S, Krobthong S, Chanroj S, Choksawangkarn W. Effects of fermentation periods on antioxidant and angiotensin I-converting enzyme inhibitory activities of peptides from fish sauce by-products. LWT. 2021;135:110122. doi: 10.1016/j.lwt.2020.110122

[40] Pownall TL, Udenigwe CC, Aluko RE. Effects of cationic property on the in vitro antioxidant activities of pea protein hydrolysate fractions. Food Res Int. 2011;44(4):1069–74. doi: 10.1016/j.foodres.2011.03.017

[41] Zhang M, Mu T-H, Sun M-J. Purification and identification of antioxidant peptides from sweet potato protein hydrolysates by Alcalase. J Func Foods. 2014;7:191–200. doi: 10.1016/j.jff.2014.02.012

[42] Aluko RE. Food protein-derived renin-inhibitory peptides: in vitro and in vivo properties. J Food Biochem. 2019;43(1):e12648. doi: 10.1111/jfbc.12648 31353494

[43] Wang S, Zhang L, Wang H, Hu Z, Xie X, Chen H, et al. Identification of novel angiotensin converting enzyme (ACE) inhibitory peptides from Pacific saury: *In vivo* antihypertensive effect and transport route. Int J Biol Macromol. 2024;254(Pt 1):127196. doi: 10.1016/j.ijbiomac.2023.127196 37793525

[44] Liu W, Chen X, Li H, Zhang J, An J, Liu X. Anti-inflammatory function of plant-derived bioactive peptides: a review. Foods. 2022;11(15):2361. doi: 10.3390/foods11152361 35954128 PMC9368234

[45] López-García J, Lehocký M, Humpolíček P, Sáha P. HaCaT keratinocytes response on antimicrobial atelocollagen substrates: extent of cytotoxicity, cell viability and proliferation. J Funct Biomater. 2014;5(2):43–57. doi: 10.3390/jfb5020043 24956439 PMC4099973

[46] Ahrens VM, Bellmann-Sickert K, Beck-Sickinger AG. Peptides and peptide conjugates: therapeutics on the upward path. Future Med Chem. 2012;4(12):1567–86. doi: 10.4155/fmc.12.76 22917246

