# Supplementary material for: Bioactivity assessment of peptides derived from salted jellyfish (Rhopilema hispidum) byproducts
Source: PLoS One. 2025 Feb 11;20(2):e0318781. doi: 10.1371/journal.pone.0318781 (PMC11813147; doi:10.1371/journal.pone.0318781)
Supplement: S1 Table — Different superscripts (A and B) in the same column mean a significant difference in value (p < 0.05). (DOCX) [file pone.0318781.s001.docx]

**S1 Table. The soluble protein content of protein hydrolysates derived from jellyfish umbrella (PU) and oral arms (PO) parts.**

| **Sample** | **Soluble protein content (mg/mL)** | |
| --- | --- | --- |
|  |  | **mean±SD** |
| **PU** | 14.04 | 14.42±0.33^A^ |
|  | 14.59 |  |
|  | 14.64 |  |
| **PO** | 13.19 | 13.64±0.45^B^ |
|  | 13.64 |  |
|  | 14.09 |  |

Different superscripts (A and B) in the same column mean a significant difference in value (p<0.05).
