# Supplementary material for: Bioactivity assessment of peptides derived from salted jellyfish (Rhopilema hispidum) byproducts
Source: PLoS One. 2025 Feb 11;20(2):e0318781. doi: 10.1371/journal.pone.0318781 (PMC11813147; doi:10.1371/journal.pone.0318781)
Supplement: S2 Table — Different superscripts (A and B) in the same column mean a significant difference in value (p < 0.05). ns = Not significant (p < 0.05). (DOCX) [file pone.0318781.s002.docx]

**S2 Table. The antioxidant activity (DPPH, ABTS, FRAP) of PU and PO.**

| **Sample** | **Antioxidant activity** | | | | | |
| --- | --- | --- | --- | --- | --- | --- |
|  | **DPPH**  **(TE/mg protein)** | | **ABTS**  **(TE/mg protein)** | | **FRAP**  **(mM FeSO_4_/mg protein)** | |
|  |  | **mean±SD** |  | **mean±SD^ns^** |  | **mean±SD** |
| **PU** | 1.89 | 1.85±0.05^A^ | 7.26 | 7.28±0.03 | 2.95 | 3.04±0.12^A^ |
|  | 1.89 |  | 7.32 |  | 3.01 |  |
|  | 1.79 |  | 7.26 |  | 3.18 |  |
| **PO** | 1.57 | 1.57±0.10^B^ | 7.33 | 7.23±0.12 | 2.19 | 2.05±0.15^B^ |
|  | 1.46 |  | 7.09 |  | 1.88 |  |
|  | 1.68 |  | 7.27 |  | 2.07 |  |

Different superscripts (A and B) in the same column mean a significant difference in value (p<0.05). ns = Not significant (p<0.05).
