# Supplementary material for: Bioactivity assessment of peptides derived from salted jellyfish (Rhopilema hispidum) byproducts
Source: PLoS One. 2025 Feb 11;20(2):e0318781. doi: 10.1371/journal.pone.0318781 (PMC11813147; doi:10.1371/journal.pone.0318781)
Supplement: S3 Table — Different superscripts (A and B) in the same column mean a significant difference in value (p < 0.05). (DOCX) [file pone.0318781.s003.docx]

**S3 Table. The antioxidant activity (DPPH, ABTS, FRAP) of PUR and POR.**

| **Sample** | **Antioxidant activity** | | | | | |
| --- | --- | --- | --- | --- | --- | --- |
|  | **DPPH**  **(TE/mg protein)** | | **ABTS**  **(TE/mg protein)** | | **FRAP**  **(mM FeSO_4_/mg protein)** | |
|  |  | **mean±SD** |  | **mean±SD** |  | **means±SD** |
| **PUR** | 3.60 | 3.60±0.30^A^ | 11.48 | 11.10±0.33^A^ | 2.94 | 3.35±0.35^A^ |
|  | 3.91 |  | 10.91 |  | 3.55 |  |
|  | 3.30 |  | 10.91 |  | 3.55 |  |
| **POR** | 2.62 | 2.51±0.18^B^ | 9.45 | 9.10±0.31^B^ | 2.51 | 2.58±0.33^B^ |
|  | 2.29 |  | 8.83 |  | 2.94 |  |
|  | 2.62 |  | 9.04 |  | 2.29 |  |

Different superscripts (A and B) in the same column mean a significant difference in value (p<0.05).
