# Supplementary material for: Bioactivity assessment of peptides derived from salted jellyfish (Rhopilema hispidum) byproducts
Source: PLoS One. 2025 Feb 11;20(2):e0318781. doi: 10.1371/journal.pone.0318781 (PMC11813147; doi:10.1371/journal.pone.0318781)
Supplement: S4 Table — Different superscripts (A, B, C, D, and E) in the same column mean significant difference in value (p < 0.05). Different superscripts (a and b) in the same row mean significant difference in value (p < 0.05). ns = Not significant (p < 0.05). (DOCX) [file pone.0318781.s004.docx]

**S4 Table. The soluble protein content of PUR10-PUR50 and POR10-POR50.**

| **Sample** | **Soluble protein content (mg/mL)** | | | |
| --- | --- | --- | --- | --- |
|  | **PUR** | | **POR** | |
|  |  | **mean±SD** |  | **mean±SD** |
| **10%ACN** | 0.98 | 1.03±0.04^Ca^ | 0.88 | 0.92±0.04^Cb^ |
|  | 1.04 |  | 0.97 |  |
|  | 1.06 |  | 0.91 |  |
| **20%ACN^ns^** | 2.13 | 2.09±0.14^A^ | 2.11 | 2.08±0.09^A^ |
|  | 2.21 |  | 2.16 |  |
|  | 1.92 |  | 1.98 |  |
| **30%ACN^ns^** | 1.46 | 1.42±0.04^B^ | 1.38 | 1.38±0.01^B^ |
|  | 1.43 |  | 1.39 |  |
|  | 1.36 |  | 1.37 |  |
| **40%ACN^ns^** | 0.39 | 0.37±0.02^D^ | 0.45 | 0.40±0.10^D^ |
|  | 0.33 |  | 0.48 |  |
|  | 0.38 |  | 0.29 |  |
| **50%ACN** | 0.01 | 0.01±0.00^Eb^ | 0.03 | 0.05±0.01^Ea^ |
|  | 0.01 |  | 0.05 |  |
|  | 0.01 |  | 0.07 |  |

Different superscripts (A, B, C, D, and E) in the same column mean significant difference in value (p<0.05). Different superscripts (a and b) in the same row mean significant difference in value (p<0.05). ns = Not significant (p<0.05).
