# Supplementary material for: Bioactivity assessment of peptides derived from salted jellyfish (Rhopilema hispidum) byproducts
Source: PLoS One. 2025 Feb 11;20(2):e0318781. doi: 10.1371/journal.pone.0318781 (PMC11813147; doi:10.1371/journal.pone.0318781)
Supplement: S5 Table — Different superscripts (A, B, C, and D) in the same column mean a significant difference in value (p < 0.05). ACN = Acetonitrile. (DOCX) [file pone.0318781.s005.docx]

**S5 Table. The antioxidant activity (DPPH, ABTS, FRAP) of jellyfish peptide after purification by reversed-phase chromatography (10–30% acetonitrile = eluent).**

| **Sample** | **Antioxidant activity** | | | | | |
| --- | --- | --- | --- | --- | --- | --- |
|  | **DPPH**  **(TE/mg protein)** | | **ABTS**  **(TE/mg protein)** | | **FRAP**  **(mM FeSO_4_/mg protein)** | |
|  |  | **mean±SD** |  | **mean±SD** |  | **mean±SD** |
| **PUR10** | 3.46 | 3.15±0.30^BC^ | 10.31 | 10.10±0.20^B^ | 2.51 | 2.58±0.12^C^ |
|  | 2.85 |  | 9.90 |  | 2.72 |  |
|  | 3.15 |  | 10.10 |  | 2.51 |  |
| **POR10** | 2.92 | 2.69±0.39^C^ | 9.26 | 9.37±0.11^C^ | 2.09 | 2.30±0.21^C^ |
|  | 2.24 |  | 9.49 |  | 2.51 |  |
|  | 2.92 |  | 9.37 |  | 2.30 |  |
| **PUR20** | 4.70 | 5.10±0.37^A^ | 13.30 | 13.17±0.33^A^ | 5.25 | 5.39±0.64^A^ |
|  | 5.15 |  | 13.41 |  | 6.09 |  |
|  | 5.45 |  | 12.79 |  | 4.83 |  |
| **POR20** | 3.52 | 3.52±0.30^B^ | 10.07 | 10.26±0.17^B^ | 3.99 | 3.92±0.94^B^ |
|  | 3.82 |  | 10.41 |  | 4.83 |  |
|  | 3.22 |  | 10.30 |  | 2.93 |  |
| **PUR30** | 3.22 | 3.08±0.12^BC^ | 10.10 | 10.14±0.05^B^ | 2.72 | 2.44±0.24^C^ |
|  | 3.00 |  | 10.21 |  | 2.30 |  |
|  | 3.00 |  | 10.10 |  | 2.30 |  |
| **POR30** | 1.51 | 1.89±0.34^D^ | 9.14 | 9.30±0.13^C^ | 2.09 | 1.74±0.32^C^ |
|  | 2.20 |  | 9.37 |  | 1.46 |  |
|  | 1.97 |  | 3.97 |  | 1.67 |  |

Different superscripts (A, B, C, and D) in the same column mean a significant difference in value (p<0.05). ACN = Acetonitrile
