# Supplementary material for: Bioactivity assessment of peptides derived from salted jellyfish (Rhopilema hispidum) byproducts
Source: PLoS One. 2025 Feb 11;20(2):e0318781. doi: 10.1371/journal.pone.0318781 (PMC11813147; doi:10.1371/journal.pone.0318781)
Supplement: S6 Table — Different superscripts (A, B, C, and D) in the same column mean a significant difference in value (p < 0.05). (DOCX) [file pone.0318781.s006.docx]

**S6 Table. The antioxidant activity (DPPH, ABTS, FRAP) of jellyfish peptide after purification by ion exchange chromatography (cation and anion exchange chromatography) and reverse phase chromatography (C18 column).**

| **Sample** | **Antioxidant activity** | | | | | |
| --- | --- | --- | --- | --- | --- | --- |
|  | **DPPH**  **(TE/mg protein)** | | **ABTS**  **(TE/mg protein)** | | **FRAP**  **(mM FeSO_4_/mg protein)** | |
|  |  | **mean±SD** |  | **mean±SD** |  | **mean±SD** |
| **PUR20C** | 5.59 | 5.52±0.06^A^ | 14.03 | 14.17±0.11^A^ | 1.88 | 1.81±0.12^C^ |
|  | 5.48 |  | 14.24 |  | 1.67 |  |
|  | 5.48 |  | 14.24 |  | 1.88 |  |
| **POR20C** | 3.52 | 3.66±0.17^B^ | 11.17 | 11.03±0.42^B^ | 0.62 | 0.76±0.12^D^ |
|  | 3.85 |  | 11.38 |  | 0.83 |  |
|  | 3.63 |  | 10.56 |  | 0.83 |  |
| **PUR20A** | 2.66 | 2.48±0.31^C^ | 6.67 | 7.15±0.42^C^ | 2.93 | 2.86±0.12^A^ |
|  | 2.11 |  | 7.49 |  | 2.72 |  |
|  | 2.66 |  | 7.28 |  | 2.93 |  |
| **POR20A** | 1.43 | 1.36±0.11^D^ | 5.03 | 5.58±0.47^D^ | 2.09 | 2.23±0.12^B^ |
|  | 1.43 |  | 5.85 |  | 2.30 |  |
|  | 1.23 |  | 5.85 |  | 2.30 |  |

Different superscripts (A, B, C, and D) in the same column mean a significant difference in value (p<0.05).
