# Supplementary material for: Bioactivity assessment of peptides derived from salted jellyfish (Rhopilema hispidum) byproducts
Source: PLoS One. 2025 Feb 11;20(2):e0318781. doi: 10.1371/journal.pone.0318781 (PMC11813147; doi:10.1371/journal.pone.0318781)
Supplement: S7 Table — Different superscripts (A, B, C, D, E, F, G, H, I, J, K, L, and M) in the same column mean a significant difference in value (p < 0.05). (DOCX) [file pone.0318781.s007.docx]

**S7 Table. The antioxidant activity (DPPH, ABTS, FRAP) of synthetic peptides (P1-P18).**

| **Sample** | **Antioxidant activity** | | | | | |
| --- | --- | --- | --- | --- | --- | --- |
|  | **DPPH**  **(TE/mg protein)** | | **ABTS**  **(TE/mg protein)** | | **FRAP**  **(mM FeSO_4_/mg protein)** | |
|  |  | **mean±SD** |  | **mean±SD** |  | **mean±SD** |
| **P1** | 0.99 | 1.16±0.15^H^ | 4.91 | 4.44±0.41^JK^ | 8.47 | 8.61±0.12^E^ |
|  | 1.25 |  | 4.11 |  | 8.68 |  |
|  | 1.25 |  | 4.31 |  | 8.68 |  |
| **P2** | 2.31 | 2.84±0.45^EFG^ | 3.11 | 2.91±0.20^KL^ | 7.21 | 7.63±0.42^EFG^ |
|  | 3.11 |  | 2.71 |  | 7.63 |  |
|  | 3.11 |  | 2.91 |  | 8.05 |  |
| **P3** | 2.84 | 2.93±0.40^EFG^ | 3.31 | 3.17±0.41^JKL^ | 9.31 | 9.24±0.12^DE^ |
|  | 2.58 |  | 2.71 |  | 9.31 |  |
|  | 3.37 |  | 3.51 |  | 9.10 |  |
| **P4** | 3.64 | 3.81±0.30^E^ | 103.28 | 103.41±1.20^F^ | 8.89 | 8.40±0.52^EF^ |
|  | 3.64 |  | 102.27 |  | 8.47 |  |
|  | 4.17 |  | 104.68 |  | 7.84 |  |
| **P5** | 2.84 | 3.20±0.40^EF^ | 124.11 | 123.24±0.75^C^ | 7.63 | 8.19±1.15^EF^ |
|  | 3.11 |  | 122.71 |  | 9.52 |  |
|  | 3.64 |  | 122.91 |  | 7.42 |  |
| **P6** | 3.11 | 3.28±0.55^EF^ | 139.34 | 139.87±0.50^A^ | 10.15 | 10.78±0.63^D^ |
|  | 2.84 |  | 139.94 |  | 11.42 |  |
|  | 3.90 |  | 140.34 |  | 10.78 |  |
| **P7** | 1.52 | 1.96±0.55^GH^ | 1.71 | 1.64±0.11^LM^ | 11.00 | 12.40±1.36^C^ |
|  | 1.78 |  | 1.71 |  | 13.73 |  |
|  | 2.58 |  | 1.50 |  | 12.47 |  |
| **P8** | 1.78 | 2.22±0.76^GH^ | 2.51 | 3.11±0.53^JKL^ | 9.31 | 9.31±0.21^DE^ |
|  | 1.78 |  | 3.31 |  | 9.52 |  |
|  | 3.11 |  | 3.51 |  | 9.10 |  |
| **P9** | 40.24 | 40.86±0.85^C^ | 105.08 | 103.21±2.27^F^ | 13.31 | 13.03±0.8^C^ |
|  | 41.83 |  | 100.67 |  | 13.73 |  |
|  | 40.51 |  | 103.88 |  | 12.05 |  |
| **P10** | 46.34 | 45.99±1.10^B^ | 97.47 | 98.00±0.50^G^ | 20.05 | 19.84±1.38^A^ |
|  | 44.75 |  | 98.07 |  | 21.10 |  |
|  | 46.87 |  | 98.47 |  | 18.36 |  |
| **P11** | 3.90 | 3.81±0.40^E^ | 4.11 | 3.91±0.20^JK^ | 6.36 | 6.36±0.63^G^ |
|  | 3.37 |  | 3.71 |  | 7.00 |  |
|  | 4.17 |  | 3.91 |  | 5.73 |  |
| **P12** | 2.58 | 2.75±0.30^EFG^ | 0.90 | 0.64±0.30^M^ | 16.26 | 16.75±0.52^B^ |
|  | 2.58 |  | 0.30 |  | 17.31 |  |
|  | 3.11 |  | 0.70 |  | 16.68 |  |
| **P13** | 5.23 | 5.85±0.66^D^ | 5.91 | 6.51±0.87^H^ | 4.89 | 4.82±0.73^H^ |
|  | 5.76 |  | 6.11 |  | 5.52 |  |
|  | 6.56 |  | 7.51 |  | 4.05 |  |
| **P14** | 6.29 | 6.29±0.53^D^ | 118.70 | 119.57±1.33^E^ | 10.36 | 9.24±1.05^DE^ |
|  | 5.76 |  | 118.90 |  | 9.10 |  |
|  | 6.82 |  | 121.11 |  | 8.26 |  |
| **P15** | 5.49 | 6.20±1.00^D^ | 132.32 | 131.66±1.15^B^ | 3.84 | 4.61±0.73^H^ |
|  | 5.76 |  | 130.32 |  | 4.68 |  |
|  | 7.35 |  | 132.32 |  | 5.31 |  |
| **P16** | 55.89 | 56.07±0.30^A^ | 121.71 | 121.31±0.87^D^ | 8.05 | 6.92±1.59^FG^ |
|  | 55.89 |  | 120.30 |  | 7.63 |  |
|  | 56.42 |  | 121.91 |  | 5.10 |  |
| **P17** | 4.70 | 5.23±0.53^D^ | 4.11 | 4.64±0.92^IJ^ | 7.63 | 6.50±1.59^G^ |
|  | 5.23 |  | 4.11 |  | 7.21 |  |
|  | 5.76 |  | 5.71 |  | 4.68 |  |
| **P18** | 5.23 | 6.03±0.79^D^ | 5.71 | 6.05±0.75^HI^ | 4.89 | 4.40±0.43^H^ |
|  | 6.03 |  | 5.51 |  | 4.05 |  |
|  | 6.82 |  | 6.91 |  | 4.26 |  |

Different superscripts (A, B, C, D, E, F, G, H, I, J, K, L, and M) in the same column mean a significant difference in value (p<0.05).
