# Supplementary material for: Bioactivity assessment of peptides derived from salted jellyfish (Rhopilema hispidum) byproducts
Source: PLoS One. 2025 Feb 11;20(2):e0318781. doi: 10.1371/journal.pone.0318781 (PMC11813147; doi:10.1371/journal.pone.0318781)
Supplement: S8 Table — Different superscripts (A, B, C, D, E, F, G, H, I, J, and K) in the same column mean a significant difference in value (p < 0.05). (DOCX) [file pone.0318781.s008.docx]

**S8 Table. The ACE inhibitory activity of synthetic peptides (P1-P18).**

| **Sample** | **ACE inhibitory activity (%)** | |
| --- | --- | --- |
|  |  | **mean±SD** |
| **Ramipril** | 22.79 | 23.23±0.44^G^ |
|  | 23.23 |  |
|  | 23.67 |  |
| **P1** | 6.67 | 6.48±0.33^IJ^ |
|  | 6.67 |  |
|  | 6.10 |  |
| **P2** | 0.66 | 0.85±0.33^K^ |
|  | 1.24 |  |
|  | 0.66 |  |
| **P3** | 33.58 | 33.87±0.28^E^ |
|  | 33.87 |  |
|  | 34.16 |  |
| **P4** | 5.53 | 5.15±0.92^J^ |
|  | 4.10 |  |
|  | 5.82 |  |
| **P5** | 24.71 | 29.10±3.81^F^ |
|  | 31.01 |  |
|  | 31.58 |  |
| **P6** | 33.87 | 33.68±0.59^E^ |
|  | 33.01 |  |
|  | 34.16 |  |
| **P7** | 8.11 | 8.68±0.49^IJ^ |
|  | 8.96 |  |
|  | 8.96 |  |
| **P8** | 32.44 | 29.96±3.35^F^ |
|  | 26.14 |  |
|  | 31.29 |  |
| **P9** | 5.53 | 7.53±1.74^IJ^ |
|  | 8.68 |  |
|  | 8.39 |  |
| **P10** | 11.25 | 9.82±1.43^HI^ |
|  | 9.82 |  |
|  | 8.39 |  |
| **P11** | 19.27 | 22.90±3.15^G^ |
|  | 25.00 |  |
|  | 24.42 |  |
| **P12** | 49.61 | 47.51±3.39^D^ |
|  | 43.60 |  |
|  | 49.33 |  |
| **P13** | 21.56 | 20.51±1.81^G^ |
|  | 18.41 |  |
|  | 21.56 |  |
| **P14** | 81.67 | 81.87±0.33^B^ |
|  | 81.67 |  |
|  | 82.25 |  |
| **P15** | 57.63 | 55.43±3.55^C^ |
|  | 51.33 |  |
|  | 57.34 |  |
| **P16** | 92.27 | 91.69±1.78^A^ |
|  | 89.69 |  |
|  | 93.12 |  |
| **P17** | 9.82 | 12.40±2.23^H^ |
|  | 13.54 |  |
|  | 13.83 |  |
| **P18** | 20.13 | 22.80±2.33^G^ |
|  | 23.85 |  |
|  | 24.42 |  |

Different superscripts (A, B, C, D, E, F, G, H, I, J, and K) in the same column mean a significant difference in value (p<0.05).
