# Supplementary material for: Bioactivity assessment of peptides derived from salted jellyfish (Rhopilema hispidum) byproducts
Source: PLoS One. 2025 Feb 11;20(2):e0318781. doi: 10.1371/journal.pone.0318781 (PMC11813147; doi:10.1371/journal.pone.0318781)
Supplement: S9 Table — Different superscripts (A, B, C, D, E, F, and G) in the same column mean a significant difference in value (p < 0.05). (DOCX) [file pone.0318781.s009.docx]

**S9 Table. The anti-inflammatory activity of synthetic peptides (P1-P18).**

| **Sample** | **Released NO (µM)** | |
| --- | --- | --- |
|  |  | **mean±SD** |
| **CM** | 0.68 | 0.45±0.39^G^ |
|  | 0.00 |  |
|  | 0.68 |  |
| **LPS** | 43.10 | 43.21±2.24^A^ |
|  | 41.03 |  |
|  | 45.51 |  |
| **P1** | 37.58 | 37.93±0.34^BC^ |
|  | 38.27 |  |
|  | 37.93 |  |
| **P2** | 38.27 | 37.70±0.52^BC^ |
|  | 37.24 |  |
|  | 37.58 |  |
| **P3** | 37.58 | 36.66±1.30^BCD^ |
|  | 35.17 |  |
|  | 37.24 |  |
| **P4** | 38.96 | 37.12±1.62^BCD^ |
|  | 35.86 |  |
|  | 36.55 |  |
| **P5** | 34.13 | 34.59±1.43^DE^ |
|  | 33.44 |  |
|  | 36.20 |  |
| **P6** | 34.82 | 36.66±2.61^BCD^ |
|  | 35.51 |  |
|  | 39.65 |  |
| **P7** | 35.51 | 35.63±1.89^CDE^ |
|  | 37.58 |  |
|  | 33.79 |  |
| **P8** | 37.58 | 36.78±1.70^BCD^ |
|  | 37.93 |  |
|  | 34.82 |  |
| **P9** | 35.86 | 35.63±0.71^CDE^ |
|  | 36.20 |  |
|  | 34.82 |  |
| **P10** | 35.86 | 36.43±1.62^BCD^ |
|  | 35.17 |  |
|  | 38.27 |  |
| **P11** | 36.55 | 36.66±0.86^BCD^ |
|  | 35.86 |  |
|  | 37.58 |  |
| **P12** | 36.89 | 38.50±1.55^B^ |
|  | 38.62 |  |
|  | 40.00 |  |
| **P13** | 35.17 | 36.09±1.30^BCDE^ |
|  | 35.51 |  |
|  | 37.58 |  |
| **P14** | 32.06 | 32.52±1.76^F^ |
|  | 31.03 |  |
|  | 34.48 |  |
| **P15** | 32.75 | 33.44±1.19^EF^ |
|  | 32.75 |  |
|  | 34.82 |  |
| **P16** | 35.17 | 34.59±0.99^DEF^ |
|  | 33.44 |  |
|  | 35.17 |  |
| **P17** | 34.48 | 34.71±0.19^DEF^ |
|  | 34.82 |  |
|  | 34.82 |  |
| **P18** | 34.13 | 32.18±1.70^F^ |
|  | 31.37 |  |
|  | 31.03 |  |

Different superscripts (A, B, C, D, E, F, and G) in the same column mean a significant difference in value (p<0.05).
