# Supplementary material for: Bioactivity assessment of peptides derived from salted jellyfish (Rhopilema hispidum) byproducts
Source: PLoS One. 2025 Feb 11;20(2):e0318781. doi: 10.1371/journal.pone.0318781 (PMC11813147; doi:10.1371/journal.pone.0318781)
Supplement: S10 Table — Different superscripts (A, B, C, D, E, F, G, and H) in the same column mean a significant difference in value (p < 0.05). UN = untreated sample. (DOCX) [file pone.0318781.s010.docx]

**S10 Table. Cytotoxicity of synthetic peptides (P1-P18).**

| **Sample** | **Cell viability (%)** | |
| --- | --- | --- |
|  |  | **mean±SD** |
| **UN** | 104.36 | 100.00±5.75^A^ |
|  | 102.15 |  |
|  | 93.47 |  |
| **LPS** | 80.66 | 83.75±2.68^CDEF^ |
|  | 85.08 |  |
|  | 85.52 |  |
| **P1** | 88.04 | 85.94±2.02^BCDEF^ |
|  | 85.76 |  |
|  | 84.00 |  |
| **P2** | 83.12 | 85.70±2.29^BCDEF^ |
|  | 86.46 |  |
|  | 87.52 |  |
| **P3** | 75.57 | 75.62±0.26^G^ |
|  | 75.92 |  |
|  | 75.39 |  |
| **P4** | 83.12 | 82.77±0.92^DEF^ |
|  | 83.47 |  |
|  | 81.72 |  |
| **P5** | 92.44 | 87.58±5.89^BCD^ |
|  | 89.27 |  |
|  | 81.01 |  |
| **P6** | 92.97 | 85.47±6.57^BCDEF^ |
|  | 82.77 |  |
|  | 80.66 |  |
| **P7** | 81.72 | 81.60±0.36^EF^ |
|  | 81.89 |  |
|  | 81.19 |  |
| **P8** | 84.35 | 83.71±1.92^CDEF^ |
|  | 85.23 |  |
|  | 81.54 |  |
| **P9** | 87.34 | 87.34±1.05^BCD^ |
|  | 86.26 |  |
|  | 88.40 |  |
| **P10** | 85.41 | 85.35±0.96^BCDEF^ |
|  | 84.35 |  |
|  | 86.29 |  |
| **P11** | 84.53 | 84.71±0.63^BCDEF^ |
|  | 85.41 |  |
|  | 84.18 |  |
| **P12** | 76.97 | 75.27±1.53^G^ |
|  | 73.98 |  |
|  | 74.86 |  |
| **P13** | 81.72 | 82.77±1.53^DEF^ |
|  | 82.07 |  |
|  | 84.53 |  |
| **P14** | 87.69 | 90.09±2.78^B^ |
|  | 89.45 |  |
|  | 93.14 |  |
| **P15** | 87.87 | 87.22±1.43^BCDE^ |
|  | 85.58 |  |
|  | 88.22 |  |
| **P16** | 81.01 | 81.19±0.17^F^ |
|  | 81.37 |  |
|  | 81.19 |  |
| **P17** | 83.47 | 83.65±0.17^CDEF^ |
|  | 83.83 |  |
|  | 83.65 |  |
| **P18** | 86.29 | 89.16±4.23^BC^ |
|  | 87.17 |  |
|  | 94.02 |  |

Different superscripts (A, B, C, D, E, F, G, and H) in the same column mean a significant difference in value (p<0.05). UN = untreated sample.
